# Transjugular Transcatheter Tricuspid Valve Replacement in Patients With Cardiac Implantable Electronic Devices

**DOI:** 10.1016/j.jacasi.2025.07.009

**Published:** 2025-08-30

**Authors:** Kent Chak-yu So, Lukas Stolz, Neil Fam, Geraldine Ong, Anson Cheung, Robert Boone, Pedro Villablanca, Ahmad Jabri, Yat-yin Lam, Didier Tchétché, Omar Oliva, Ole De Backer, Jacob Eifer Mølller, Azeem Latib, Andrea Scotti, Augustin Coisne, Arnaud Sudre, Julien Dreyfus, Mohammed Nejjari, Paul-Emile Favre, Ignacio Cruz-Gonzalez, Rodrigo Estévez-Loureiro, Manuel Barreiro-Perez, Raj Makkar, Dhairya Patel, Guillaume Leurent, Erwan Donal, Thomas Modine, Jörg Hausleiter

**Affiliations:** a Division of Cardiology, Department of Medicine and Therapeutics, Prince of Wales Hospital, Chinese University of Hong Kong, Hong Kong Special Administration Region, China; b Medizinische Klinik und Poliklinik I, Ludwig-Maximilians University Klinikum, Ludwig-Maximilians University München, Munich, Germany; c St Michael’s Hospital, University of Toronto, Toronto, Ontario, Canada; d Centre for Cardiovascular Innovation, St Paul's and Vancouver General Hospital, Vancouver, British Columbia, Canada; e Henry Ford Hospital, Detroit, Michigan, USA; f Clinique Pasteur, Toulouse, France; g Department of Cardiology, The Heart Center, Rigshospitalet, University of Copenhagen, Copenhagen, Denmark; h Montefiore-Einstein Center for Heart and Vascular Care, Montefiore Medical Center, Albert Einstein College of Medicine, Bronx, New York, USA; i Universitaire Lille, Inserm, Centre Hospitalier Universitaire Lille, Institut Pasteur de Lille, Unite 1011-European Genomic Institute for Diabetes, Lille, France; j Cardiology Department, Centre Cardiologique du Nord, Saint-Denis, France; k Department of Cardiology and Cardiovascular Surgery, Heart Valve Center, Institut Cœur Poumon Centre Hospitalier Universitaire de Bordeaux, France; l Department of Cardiology, Complejo Asistencial Universitario de Salamanca, Salamanca, Spain; m Department of Interventional Cardiology, Hospital Alvaro Cunqueiro, Vigo, Spain; n Instituto de Investigación Galicia Sur, Servizo Galego de Saude, Vigo, Spain; o Department of Cardiology, Smidt Heart Institute, Cedars-Sinai Medical Center, Los Angeles, California, USA; p Department of Cardiology, Centre Hospitalier Universitaire Rennes, Inserm, Laboratoire Traitement du Signal et de l-Image-Unité Mixte de Recherche 1099, Universitaire Rennes 1, Rennes, France; q German Center for Cardiovascular Research, Partner Site Munich Heart Alliance, Munich, Germany

**Keywords:** cardiac implantable electronic device, transcatheter lead extraction, transcatheter tricuspid valve replacement, tricuspid regurgitation

## Abstract

**Background:**

Cardiac implantable electronic device (CIED)-related tricuspid regurgitation (TR) is common. Transcatheter tricuspid valve replacement (TTVR) is feasible with CIEDs in the right ventricle; however, data in this population are limited.

**Objectives:**

This study retrospectively analyzed patients undergoing compassionate-use transjugular TTVR with the LuX-Valve Plus for symptomatic TR with CIEDs from January 2022 to August 2024 at 17 international centers.

**Methods:**

The primary endpoint was procedural TR reduction. Secondary endpoints included TR reduction, survival at 30 days, NYHA functional class changes, and CIED function at follow-up. Non-CIED group was used for comparison.

**Results:**

Of 99 patients, 36 (36.4%) had CIEDs. Baseline characteristics were similar, though the CIED group had a higher EuroSCORE (European System for Cardiac Operative Risk Evaluation) II score and more comorbidities. Procedural success (CIED vs non-CIED: 91.7% vs 95.2%; *P* = 0.781), 30-day mortality (5.6% vs 4.8%; *P* > 0.999), TR reduction (≤1+: 83.8% vs 84.9%; *P* > 0.999), and NYHA functional class I/II (80.8% vs 83.7%; *P* = 0.89) were comparable. The CIED cohort exhibited a higher numerical incidence of conversion to surgery (8.3% vs 1.6%) and tricuspid reintervention (11.5% vs 3.3%) within 6 months; however, these differences did not reach statistical significance (*P* = 0.267 and *P* = 0.160, respectively). Of the 22 patients with postoperative interrogation (median of 3.3 months), 9.1% of CIED patients exhibited worsening device parameters, with no need for lead replacement or extraction.

**Conclusions:**

Transjugular TTVR is safe and effective for managing TR and heart failure in patients with CIEDs. Due to the small sample size, these findings highlight the need for larger, prospective studies to validate these outcomes.

Cardiac implantable electronic devices (CIEDs) play a crucial role in managing patients with bradycardia, arrhythmia, and heart failure. Patients with CIED demonstrate a higher incidence of tricuspid regurgitation (TR) than the general population,^1^, ^2^, ^3^ due to various causes, including direct leaflet damage, mechanical lead tethering, impingement, or pacing-induced changes to right ventricular (RV) geometry.^4^, ^5^, ^6^, ^7^, ^8^, ^9^ Despite the recent advancement in percutaneous tricuspid valve (TV) interventions, management decisions for patients with CIEDs and symptomatic, severe TR are complex. Transvenous lead extraction (TLE) may either worsen or improve TR severity.^10^ The presence of RV leads can make tricuspid transcatheter edge-to-edge repair (T-TEER) challenging. On the other hand, “jailing” a CIED lead during transcatheter tricuspid valve replacement (TTVR) could potentially result to lead displacement, dysfunction, or damage during valve manipulation, and pose a management challenge in cases of lead-related infection.^10^

Initial findings of TTVR using SAPIEN (Edwards Lifesciences) or Evoque (Edwards Lifesciences) with CIED leads in place are mixed, posing unresolved concerns on long-term lead dysfunction.^11^, ^12^, ^13^ Besides, the risk of jailing a RV lead could be device-specific given the different anchor and valve designs, radial force, and oversizing strategy. The LuX valve Plus (Jenscare Biotechnology Co Ltd) is a radial force-independent transjugular TTVR device and is shown to be a safe and effective treatment option for patients with severe TR even in very large tricuspid anatomies.^14^^,^^15^ This study aimed to retrospectively analyze the procedural and follow-up outcomes of patients who underwent the transjugular TTVR for symptomatic TR while having CIED leads in place.

## Methods

This analysis was conducted across 17 international centers between January 1, 2022 and August 31, 2024 (Supplemental Table 1). A detailed retrospective review was conducted on patients who received the transjugular TTVR. Patients with CIEDs were analyzed, and those without CIEDs were included for comparison. The data collection method has been previously described.^14^ In brief, patients were treated after approval from the corresponding regulatory board under compassionate use determined by multidisciplinary heart team. Preoperative echocardiogram and computed tomography were evaluated for the transjugular TTVR. The procedure was performed as previously described^15^ under fluoroscopy and transesophageal echocardiogram guidance. All clinical and echocardiographic data are site-reported. The collected clinical baseline characteristics included demographic, laboratory, relevant comorbidities and echocardiographic data. The assessment of heart failure symptoms comprised NYHA functional class, peripheral edema, pleural effusion, and ascites. Important CIED parameters collected include type of CIED, number of RV leads, pacing threshold, lead sensitivity, and percentage of RV lead pacing requirement at baseline and at follow-up. Important echocardiographic parameters collected include severity of TR, left ventricular and RV function at baseline and at follow-up, location of RV lead relative to the TV, and location and severity of paravalvular leak (PVL) at follow-up. The study was approved by the Joint Chinese University of Hong Kong–New Territories East Cluster Clinical Research Ethic Committee, and the analysis was conducted with the principles outlined in the Declaration of Helsinki.

### Endpoints

The primary endpoints were intraprocedural success according to the Tricuspid Valve Academic Research Consortium (TVARC) consensus statement,^16^ TR reduction at postprocedure and at 1-month follow-up. In this context, intraprocedural success is defined as the successful implantation and precise positioning of the intended device without the need for unplanned additional devices, resulting in adequate device performance—specifically, reduction of TR to moderate severity (≤2+) or less—and the absence of major procedural complications. These complications include intraprocedural mortality or stroke, device-related pulmonary embolism, emergency surgery, or reintervention within the first 24 hours related to the device or access procedure.^16^ The secondary endpoints included procedure time, procedural complications, need for unplanned in-hospital open-heart surgery, in-hospital death, major bleeding complications, myocardial infarction, stroke, new-onset renal failure requiring dialysis, and conduction disturbance requiring new pacemaker implantation. Thirty-day clinical success was assessed according to TVARC criteria, which require correct device positioning with adequate performance, and the absence of procedural mortality, stroke, unplanned surgical or interventional procedures related to the device, or major device- or procedure-related serious adverse events.^16^ Additionally, the severity of PVL, heart failure symptoms (NYHA functional class), need for repeat TV intervention, CIED function, requirement for RV lead reprogramming, replacement, or extraction, and survival at 1-month and at the latest follow-up were assessed. The association between any PVL and severe PVL with symptom burden at follow-up was examined to assess the clinical implications of PVL.

### Statistical analysis

Parametric data are presented as mean ± SD, and nonparametric data are presented as median (Q1-Q3), and differences were determined by 2-sample Student’s *t*-test or Mann-Whitney *U* test, respectively. Pacing parameters before and after the operation were compared using Wilcoxon tests. Outcome incidence rates were reported with 95% CIs, calculated using the Clopper-Pearson (exact) method. A 2-sided *P* value <0.05 was considered statistically significant. All analyses were performed using R (version 4.0.4, R Foundation for Statistical Computing) and SPSS (version 29, IBM Corp).

## Results

### Baseline characteristics

In this retrospective analysis, 36 of 99 patients (36.4%) had CIEDs positioned across the TV. Compared to the non-CIED group, there were no statistically significant differences in age, body mass index, percentage of atrial fibrillation, hypertension, diabetes, baseline symptom burden, TRI-SCORE (Risk score model for isolated tricuspid valve surgery), left ventricular ejection fraction, baseline TR severity, mean pulmonary artery pressure, capillary wedge pressure, or right atrial V-wave (Table 1). However, the CIED cohort had a higher EuroSCORE (European System for Cardiac Operative Risk Evaluation) II score (median: 7 [Q1-Q3: 3.9-10.5] vs 4.1 [Q1-Q3: 2.3-6.0]; *P* = 0.017), more history of prior cardiac surgery, prior stroke/transient ischemic attack, a lower baseline estimated glomerular filtration rate (41.5 ± 23.2 mL/min vs 51.7 ± 23.2 mL/min; *P* = 0.041) (Table 1).

**Table 1:** Baseline Characteristics Comparison in Transjugular TTVR Patients With and Without CIED

	CIED (n = 36)	No CIED (n = 63)	*P* Value
Demographics			
Age, y	77.4 ± 8.4	74.3 ± 9.0	0.093
Female	47.2 (17/36)	50.8 (32/63)	0.894
BMI, kg/m^2^	24.2 ±4.9	25.5 ± 4.7	0.230
Dyslipidemia	72.2 (26/36)	50.8 (32/63)	0.038
HT	80.6 (29/36)	61.9 (39/63)	0.089
Previous MI	11.1 (4/36)	9.5 (6/63)	>0.999
COPD	16.7 (6/36)	19.0 (12/63)	0.980
DM	22.2 (8/36)	20.6 (13/63)	>0.999
Prior stroke/TIA	25 (9/36)	6.3 (4/63)	0.020
History of cardiac surgery	61.1 (22/36)	39.7 (25/63)	0.040
EuroSCORE II score	7 (3.9-10.5)	4.1 (2.3-6.0)	0.017
TRI-SCORE score	6.4 ± 2.6	5.5 ± 2.5	0.160
Atrial fibrillation/atrial flutter	91.7 (33/36)	88.9 (56/63)	0.925
CAD	47.2 (17/36)	27.0 (17/63)	0.041
Prior PCI	8.3 (3/36)	11.1 (7/63)	0.925
Prior CABG	30.6 (11/36)	14.3 (9/63)	0.093
Baseline conduction abnormality	27.8 (10/36)	17.5 (11/63)	0.341
NYHA functional class, median	III	III	0.373
LVEF, %	53.7 ± 11.2	55.4 ±9.0	0.419
TR severity baseline, median	4	4	0.685
eGFR, mL/min	41.5 ± 23.2	51.7 ± 23.2	0.041
Total bilirubin, mg/dL	1.05 (0.78-2.20)	0.86 (0.61-1.19)	0.088
NT-proBNP	2,548 (702-5,231)	1,599 (1,106-3,643)	0.571
Mean pulmonary artery pressure, mm Hg	23.5 (16.5-36.0)	23.0 (19.0-27.5)	0.752
Pulmonary capillary wedge pressure, mm Hg	15.8 ± 7.7	16.3 ± 7.5	0.816
Right atrial V-wave, mm Hg	20 (18-24)	18 (12-31)	0.974
Procedure parameters			
Device size, mm			0.912
Median	55	55	
35	0	1 (1.6)	
40	3 (8.3)	3 (4.8)	
45	0	1 (1.6)	
50	8 (22.2)	12 (19.0)	
55	9 (25)	21 (33.3)	
60	6 (16.7)	15 (23.8)	
65	10 (27.8)	10 (15.9)	
Large valve (≥55 mm) used	69.4 (25/36)	73.0 (46/63)	0.704

Values are mean ± SD, % (n/N), or median (Q1-Q3) unless otherwise indicated.

BMI = body mass index; CABG = coronary artery bypass graft; CAD = coronary artery disease; CIED = cardiac implantable electronic device; COPD = chronic obstructive pulmonary disease; DM = diabetes mellitus; eGFR = estimated glomerular filtration rate; EuroSCORE = European System for Cardiac Operative Risk Evaluation; HT = hypertension; LVEF = left ventricular ejection fraction; MI = myocardial infarction; NT-proBNP = N-terminal pro–brain natriuretic peptide; PCI = percutaneous coronary intervention; TIA = transient ischemic attack; TR = tricuspid regurgitation; TTVR = transcatheter tricuspid valve replacement.

In the CIED group, the predominant type of CIEDs were conventional pacemakers, accounting for 68.6% (24 of 35) of cases, whereas defibrillator leads were present in 25.7% (9 of 35) of patients, and leadless pacemakers in 5.7% (2 of 35) (Table 2). Anatomically, 50% (11 of 22) of the RV leads were positioned at the posteroseptal commissure or posterior leaflet, whereas others were positioned at the septal leaflet (18.2%, 4 of 22), anteroseptal commissure (9.1%, 2 of 22), central (9.1%, 2 of 22), and anteroposterior commissure (4.5%, 1 of 22) positions before TTVR. The median number of RV leads per patient was 1 (ranging from 1 to 3). Only 1 case involved RV lead extraction and leadless pacemaker implantation prior to TTVR. Among these patients, only 15.4% (4 of 26) had CIED-induced TR, whereas in the remaining patients, the CIED was incidental, and the mechanisms of TR were primary (3.8%, 1 of 26), secondary (atrial or ventricular, 46.2%, 12 of 26), and mixed etiology (34.6%, 9 of 26), respectively.

**Table 2:** Features of CIEDs in Those Who Underwent the Transjugular TTVR (N = 36)

Type of CIED	
ICD	20 (7/35)
CRTD	5.7 (2/35)
Conventional pacemaker	68.6 (24/35)
Leadless pacemaker	5.7 (2/35)
Number of RV leads	1 (1-3)
Location of RV leads	
Posteroseptal commissure	50 (11/22)
Posterior leaflet	9.1 (2/22)
anteroposterior commissure	4.5 (1/22)
Anterior leaflet	0
anteroseptal commissure	9.1 (2/22)
Septal leaflet	18.2 (4/22)
Central	9.1 (2/22)

Values are % (n/N) or median (range).

CRTD = cardiac resynchronization therapy and defibrillators; ICD = implantable cardioverter defibrillator; other abbreviations as in Tables 1 and 2.

### Procedural outcome and TR reduction

Larger valve sizes (>55 mm) were more commonly used in both cohorts of patients (69.4% [25 of 36] vs 73.0% [46 of 63]; *P* = 0.704). There were no statistically significant differences in the TVARC intraprocedural success and 30-day clinical success rates between the 2 groups (CIED group vs non-CIED group: 91.7% [95% CI: 77.5%-98.2%] vs 95.2% [95% CI: 86.1%-98.6%]; *P* = 0.781; and 90.3% [95% CI: 75.8%-97.0%] vs 90.9% [95% CI: 79.4%-96.4%]; *P* > 0.999). There was no statistically significant difference in procedure time between the groups (CIED group: median: 155 [Q1-Q3: 28-164] minutes vs non-CIED group: median: 124 [Q1-Q3: 87-160] minutes; *P* = 0.108) (Table 3). The device and procedural complication rates were similar between the 2 groups (CIED group vs non-CIED group: 8.3% [95% CI: 1.7%-22.5%] vs 3.2% [95% CI: 0.4%-11.5%]; *P* = 0.350). The CIED cohort exhibited a higher numerical incidence of conversion to surgery (8.3% [95% CI: 1.7%-22.5%] vs 1.6% [95% CI: 0.04%-8.6%]); however, the differences did not reach statistical significance (*P* = 0.267). Of the 3 CIED cases, 1 patient experienced cardiac tamponade prior to valve implantation, necessitating open perforation repair; this patient survived. Another patient had device malpositioning that led to significant TR and subsequently underwent TVR but unfortunately died during hospitalization from right heart failure. The third patient had delayed valve embolization, which required TVR, and this patient also survived.

**Table 3:** Comparison Outcomes After Transjugular TTVR in Patients With and Without CIEDs

	CIED (n = 36)	No CIED (n = 63)	*P* Value
Procedure time, min	155 (128-164)	124 (87-160)	0.108
TVARC intraprocedural success	91.7 (33/36)	95.2 (60/63)	0.781
Device and procedural complications	8.3 (3/36)	3.2 (2/63)	0.350
Malposition of the device	1	0	
Embolization of the device	1	0	
Pericardial effusion before valve deployment	1	0	
Anchor detachment	0	1	
Incomplete extension of anterior leaflet graspers	0	1	
Need for in-hospital open heart surgery	8.3 (3/36)	1.6 (1/63)	0.267
Inpatient death	5.6 (2/36)	4.8 (3/63)	0.962
Right heart failure	1 (2.8)	2 (3.2)	
Gastrointestinal bleeding	1 (2.8)	0	
Tamponade	0	1 (1.6)	
In-hospital bleeding complication	5.6 (2/36)	7.9 (5/63)	>0.999
In-hospital myocardial infarction	0 (0/36)	0 (0/63)	>0.999
In-hospital stroke	0 (0/36)	0 (0/63)	>0.999
In-hospital new-onset renal failure requiring dialysis	5.6 (2/36)	0 (0/63)	0.130
Inpatient requiring new permanent pacemaker	0 (0/36)	4.8 (3/63)	0.552
30-day TVARC clinical success	90.3 (28/31)	90.6 (48/53)	>0.999
30-day death	5.6 (2/36)	4.8 (3/63)	>0.999
30-day NYHA functional class improvement ≥1 grade	76.9 (20/26)	81.6 (40/49)	0.624
30-day NYHA functional class I/II	84.6 (22/26)	87.8 (43/49)	0.704
30-day ≤ mild TR (≤1+)	83.9 (26/31)	84.9 (45/53)	>0.999
TR reduction ≥1 grade	100 (31/31)	98.1 (52/53)	>0.999
TR reduction ≥2 grade	96.8 (30/31)	98.1 (52/53)	>0.999
30-day any PVL	67.7 (21/31)	24.5 (13/53)	<0.001
30-day ≥severe PVL	9.7 (3/31)	7.5 (4/53)	0.412
Repeat TV intervention at follow-up	11.5 (3/26)	3.3 (2/60)	0.160
Heterotopic TTVR for severe PVL due to septal anchor detachment	1	0	
PVL plug	2	1	
TVR for severe PVL due to septal anchor detachment	0	1	
RV lead dysfunction requiring revision at follow-up	0 (0/36)	N/A	N/A
RV lead dysfunction requiring reprograming at follow-up	9.1 (2/22)	N/A	N/A

Values are median (Q1-Q3), % (n/N), n, or n (%).

N/A = not applicable; PVL = paravalvular leak; RV = right ventricle; TV = tricuspid valve; TVARC = Tricuspid Valve Academic Research Consortium; other abbreviations as in Table 1.

**Figure 1:**
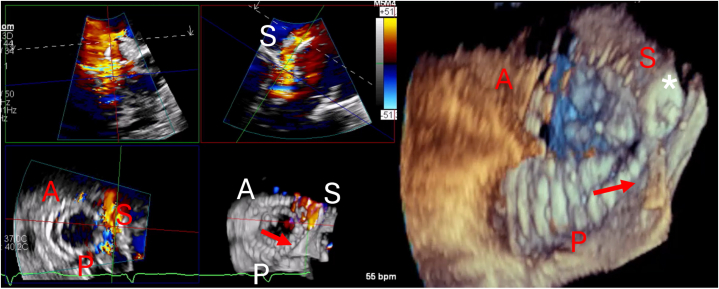
Severe PVL Near RV Lead After Transjugular TTVR (Left) Multiplanar reconstruction from 3-dimensional transesophageal echocardiogram showing septal severe paravalvular leak (PVL) adjacent to a pacemaker right ventricular (RV) lead that was located at posteroseptal commissure before transcatheter tricuspid valve replacement (TTVR) (red arrow). (Right) Successful PVL closure using a plug device (∗) reducing PVL to mild. A = anterior; P = posterior; S = septal.

The risks of 30-day mortality were similar (5.6% [95% CI: 0.7%-18.3%] vs 3.2% 95% CI: 0.4%-11.5%); *P* = 0.962). At 30 days, patients maintained ≤ mild TR (≤1+; 83.8% [95% CI: 68.1%-93.2%] vs. 84.9% [95% CI: 73.5%-92.3%]; *P* ≥ 0.999), and symptom improvement (NYHA functional class I/II: 80.8% [95% CI: 64.7%-90.9%] vs 83.7% [95% CI: 72.1%-91.1%]; *P* = 0.89) was similar (Central Illustration). In the CIED group, 5 patients experienced worsening TR from immediately after intervention to 1 month later. This was due to 1 case of septal anchor detachment and 4 cases of PVL progressing to moderate or severe levels. Similarly, 5 patients in the non-CIED group also had TR progression at 1-month postintervention that was attributed to 1 case of lost posterior leaflet grasper and 4 cases of PVL progressing to moderate or severe. Although the risk of any PVL (including mild PVL) was higher in the CIED group compared to the non-CIED group (67.7% [95% CI: 50.3%-81.8%] vs 24.5% [95% CI: 14.4%-37.5%]; *P* < 0.001), with the majority occurring next to the jailed RV lead (87.0%), the risk of severe PVL was similar (9.7% [95% CI: 2.1%-25.6%] vs 7.5% [95% CI: 2.1%-19.9%] in those without a CIED lead; *P* = 0.412). Two-thirds of the severe PVL in the CIED group occurred adjacent to the jailed lead. Overall, severe PVL was significantly associated with suboptimal symptom improvement during follow-up (NYHA functional class III/IV: 71.4% [5 of 7] vs 11.3% [8 of 71] in those without severe PVL; relative risk: 6.33; 95% CI: 2.84-14.1), but this was not observed in cases with mild or greater PVL (NYHA functional class III/IV: 25.0% [8 of 32] vs 10.9% [5 of 46] in those without severe PVL; relative risk: 2.30; 95% CI: 0.83-6.41). At 6-month follow-up, the incidence of repeat TV intervention appeared elevated in the CIED group (11.5% [95% CI: 3.2%-26.9%] vs 3.3% [95% CI: 0.4%-11.5%]), although this difference did not reach statistical significance (*P* = 0.160). In the CIED group, 2 severe PVL cases occurred next to the jailed RV lead and 1 due to septal anchor detachment. The risk of PVL showed no difference between pacemaker and defibrillator leads (9.1% [95% CI: 1.1%-29.2%] vs 11.1% [95% CI: 0.3%-48.3%]; *P* = 1.00). Apart from the case of septal anchor detachment, the remaining 2 severe PVL cases had RV leads located at posteroseptal commissure before TTVR. Among these patients, 1 underwent PVL plugging using Amplatzer Vascular Plus device (Abbott Cardiovascular) reducing PVL from severe to mild, 1 planned for PVL plugging and 1 was treated with heterotopic TTVR (Figure 1). An additional separate outcome analysis was also performed after excluding the 2 leadless pacemaker cases from the CIED cohort, which yielded similar results (Supplemental Table 2).

**Central Illustration:**
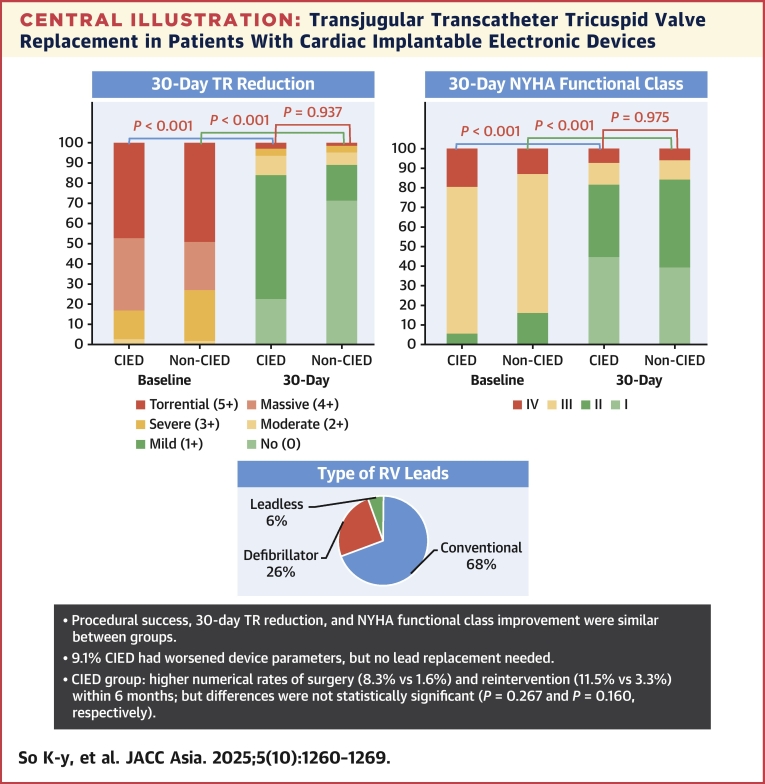
Transjugular Transcatheter Tricuspid Valve Replacement in Patients With Cardiac Implantable Electronic Devices Transjugular transcatheter tricuspid valve replacement is safe and effective in reducing tricuspid regurgitation (TR) and improve heart failure symptoms in cardiac implantable electronic device (CIED) patients. Of the 22 patients with postoperative interrogation (median: 3.3 months), 9.1% of CIED patients exhibited worsening device parameters, with no need for lead replacement or extraction. RV = right ventricular.

### CIED characteristics and function

**Figure 2:**
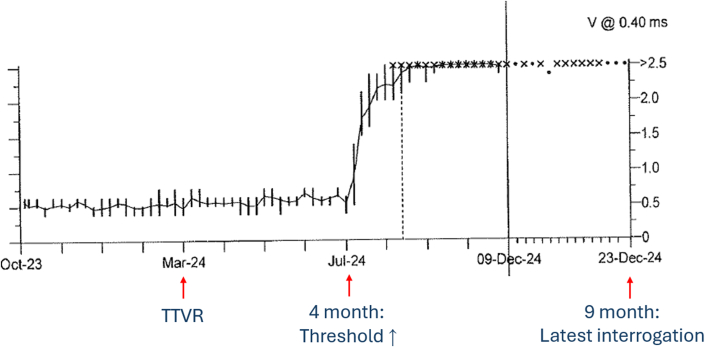
Pacing Threshold Trend in Patient With CIED and Transjugular TTVR The pacing threshold only started to increase 4 months after transcatheter tricuspid valve replacement (TTVR), which gradually progressed, and fluctuated around 2.5 V at 0.40 millisecond. Reprogramming was performed without the need of lead revision until the latest follow-up at 9 months.

**Figure 3:**
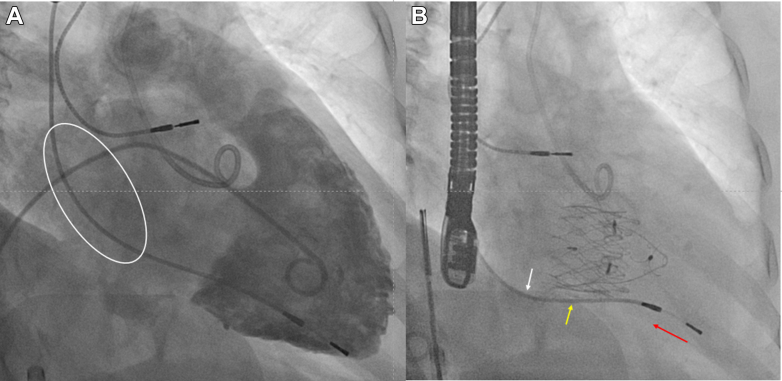
RV Lead Position Before and After TTVR (A) Fluoroscopic images showing position of the RV lead before the transjugular TTVR, showing minimal slack (white circle). (B) Several potential interactions between the transjugular TTVR and the RV leads are visible in the fluoroscopic image taken from the same projection after valve implantation, including changes in slack or tension on the RV lead (white arrow) following displacement from the septal to posterior position, direct compression of the RV lead by the valve (yellow arrow), and subtle displacement of the RV lead insertion point (red arrow). Abbreviations as in Figure 1.

After a median follow-up of 3.3 (Q1-Q3: 1.3-7.8) months, no patients required RV lead replacement or extraction. Of the 22 patients with at least 1 device interrogation result after the intervention, there were no statistically significant differences in observed lead impedance, pacing sensitivity, threshold or percentage of RV pacing before the operation and during follow-up after TTVR (Table 4). However, 2 cases (9.1%; 95% CI: 1.1%-28.7%) showed worsening of RV lead parameters. One patient with a size 60-mm valve implanted and RV lead located at the septal side experienced an increased RV lead threshold from 0.5 V at 0.4 milliseconds to 2.37 V at 0.4 milliseconds 4 months postintervention, after which it remained similar until the latest 1-year follow-up (Figures 2 and 3). The other patient with a 40-mm valve implanted and RV lead located at posteroseptal commissure had a decline in RV lead sensitivity from 11.2 to 3.9 mV immediately postintervention, which then stabilized. Both cases were managed with reprogramming of the device without need for lead revision.

**Table 4:** RV Lead Function Before and After Transjugular TTVR

	Before TTVR	Post TTVR	*P* Value
Lead impedance, Ω	485 (367-614)	445 (420-564)	0.327
Pacing sensitivity, mV	7.0 (0.9-11.2)	4.1 (0.9-14)	0.674
Pacing lead threshold, mA	0.73 (0.59-1.00)	0.60 (0.50-0.93)	0.512
RV pacing, %	72 ± 33	79 ± 30	0.905

Values are median (Q1-Q3) or mean ± SD.

Abbreviations as in Tables 1 and 2.

## Discussion

This is the largest international multicenter retrospective registry on outcomes following the transjugular TTVR in the presence of CIED for patients who were treated in a compassionate setting. This registry showed similar TR reduction and heart failure symptom improvement in patients with CIED and without CIED. Within the first one-half year after TTVR, no jailed RV leads required revision.

### Procedural outcome and TR reduction

The intraprocedural and 1-month TR reduction after the transjugular TTVR in patients with CIEDs was reasonable (91.7% and 83.8% reduced to mild, respectively), even within this cohort of compassionate use and early experience cases. These results were comparable to those from other transfemoral TTVR platforms, such as the EVOQUE TTVR system (Edwards Lifesciences), in their first-in-human compassionate use experiences (92% and 88% TR reduction to mild intraprocedurally and at 1 month).^17^ However, outcomes specific to the CIED cohort (36% of cases) were not reported in their series. In our experience, it was observed that in some patients, the PVL progressed within 1 month. One CIED case had a septal anchor detachment and the other non-CIED case had grasper loss of leaflet capture with valve tilting, resulting in massive PVL, whereas in other cases, the valve appeared stable. The progression of PVL could relate to suboptimal valve sizing, changes in the patient’s loading conditions, or subtle valve tilting during heart contractions and remodeling.

Despite these concerns, significant symptom improvements were observed post transjugular TTVR in majority of the patients with CIEDs, with 80.8% experiencing symptom relief following the procedure. Our analysis illustrated that jailing of CIED leads was associated with PVL, but most leaks were clinically insignificant (1+), with only 9.7% categorized as severe. Severe PVL was linked to suboptimal symptom improvement (relative risk: 6.3), whereas the risk of severe PVL in the CIED group was comparable to that in patients without CIED (7.5%). This risk of PVL was reported to be lower for other transfemoral TTVR devices.^17^ The discrepancies may be related to variations in valve design, such as the absence of a sealing skirt, different radial force, and the impact of 3-point anchors vs 12-point leaflet grasping on valve alignment and oversizing strategies in commercial transfemoral TTVR systems. However, it remains uncertain whether increased oversizing will indeed lead to less PVL, and less oversizing might even be beneficial in lead patients because of a lower risk for lead failure. Unfortunately, preoperative computed tomography and follow-up echocardiographic images were not available in the current study to determine the precise mechanism of PVL.

In the presence of an RV lead, valve expansion and annular contact may be compromised. Furthermore, valve alignment could be jeopardized, especially if the lead is located on the lateral side of the TV and if the RV lead has no slack to accommodate the transcatheter valve. The risk of PVL may potentially be reduced with careful simulation of valve frame interactions with pre-existing RV leads, along with increased operator experience in valve deployment, improved sizing strategies, and better patient selection in individuals with RV leads in situ.

### CIED outcome

Concerns regarding jailing an RV lead during TTVR include the risk of lead dysfunction due to the mechanical interaction between the TTVR and the jailed lead, as well as the risk of infection. In our analysis, none of the cases required RV lead revision within a median follow-up of 3.3 (Q1-Q3: 1.3-7.8) months post-TTVR. However, in 2 cases, lead parameters deteriorated after the procedure. Notably, in 1 case, the pacing threshold of the lead began to increase 4 months after TTVR. This illustrates the possibility of delayed lead dysfunction and highlights the importance of close monitoring of lead parameters following TTVR. Lead dysfunction after TTVR may occur due to direct compression of the RV lead by the valve frame or lead displacement (Figure 3).

The reported risk of RV lead dysfunction has yielded mixed results in studies on commercial transfemoral TTVR. In a multicenter first-in-human series, the reported rate was 0,^12^ while a smaller single-center series reported a high incidence of lead malfunction related to RV lead jailing during TTVR.^13^ Comparatively, the transjugular TTVR included in this analysis involved larger devices, potentially resulting in more significant displacement of RV leads after valve implantation. The transjugular TTVR features an asymmetrical valve design with a large lateral atrial skirt, and theoretically, an RV lead with a lateral trajectory may be more significantly displaced, interfering with valve alignment. However, this was not observed in our series, likely due to the preoperative exclusion of unfavorable cases.

Moreover, aside from case selection, the reasonable outcomes of transjugular TTVR in patients with CIEDs could also be associated with the radial force-free anchor mechanism provided by the 2 graspers and a septal anchor hook. This design enhances valve stability while minimizing oversizing relative to the tricuspid annulus and reducing compression on the conduction system. Consequently, this feature has resulted in a lower rate of heart block requiring permanent pacemakers^14^; however, whether this reduction also decreases the risk of lead dysfunction remains uncertain. Future digital simulations to guide case selection may improve CIED outcomes in TTVR.

### Undefined optimal approach

As CIED-related TR becomes more recognized, it remains unclear which interventional approach is the most effective. Recent data suggest that T-TEER effectively reduces TR in patients with CIEDs in situ and has limited impact on RV lead function.^18^^,^^19^ However, these patients are often highly selected with favorable anatomy for T-TEER, whereas our cohort consisted of patients with unfavorable anatomy for T-TEER, such as a wide coaptation gap and leaflet impingement by CIED leads. Therefore, a direct comparison is not valid. TLE is rarely performed to treat TR.^2^ In our cohort, only 1 patient underwent TLE with a leadless pacemaker inserted before TTVR. Whether this is the optimal approach remains uncertain. CIED-related TR is complex due to mixed pathology, and an individualized approach is likely needed, considering the location of RV leads, years of implantation, the mechanism of TR, and TV anatomy. Thus, a multidisciplinary approach involving electrophysiologists is recommended. Additionally, more head-to-head comparisons of T-TEER, different TTVR platforms, and TLE in selected patient cohorts will be necessary.

### Study limitations

First, the study is retrospective; however, it includes the largest cohort focusing on TTVR in patients with pre-existing CIEDs. Having said that, due to the overall limited sample size, the findings were exploratory and need to be interpreted with caution. Second, the study included patients under a compassionate use protocol only, in which TR was usually at an advanced stage; therefore, the same conclusions may not be generalized to patients with earlier stages of TR. Third, there was no core-lab adjudication, but all site-reported data were collected by experienced physicians and echocardiographers at each study center according to recent guidelines and recommendations. Fourth, important data, including preprocedural computed tomography, some hemodynamic data such as pulmonary vascular resistance, and functional outcomes such as the 6-minute walk test or quality of life questionnaires, were not available, which limited the potential for mechanistic understanding of the interaction between the RV lead and TTVR.

## Conclusions

This study demonstrated similar TR reduction and heart failure symptom improvement following TTVR in patients with CIEDs and those without CIEDs, with no lead revisions required during follow-up. Longer-term follow-up, larger scale prospective data will be needed to confirm these findings.

## Funding Support and Author Disclosures

Dr So has served as a proctor for Abbott, Boston Scientific, Edwards Lifesciences, and Medtronic; and as a consultant for Venus Medtech and Jenscare. Dr Stolz has received speaker honoraria from Edwards Lifesciences. Dr Fam has served as a consultant for Edwards Lifesciences, Abbott, and Cardiovalve. Dr Cheung has received speaker honoraria from Edwards Lifesciences, Abbott Vascular, and Medtronic; and has served as an eligibility committee member for the TRINITY trial. Dr Boone has served as a consultant for Edwards Lifesciences and Abbott. Dr Villablanca has served as a consultant for Edwards Lifesciences, Medtronic, Angiodynamic, Telflex, and Abiomed. Dr Tchétché has served as a consultant for Abbott, Edwards Lifesciences, Boston Scientific, and Medtronic. Dr De Backer has received institutional research grants and consulting fees from Abbott, Boston Scientific, and Medtronic. Dr Latib has served on the Advisory Board for Medtronic, Abbott Vascular, Boston Scientific, Edwards Lifesciences, Shifamed, NeoChord Inc, V-dyne, and Philips. Dr Scotti has served as a consultant for NeoChord and Edwards Lifesciences. Dr Dreyfus has received proctoring or consulting fees from Abbott and Edwards Lifesciences. Dr Estévez-Loureiro has served as a consultant to Abbott Vascular, Edwards Lifesciences, Boston Scientific, Venus Medtech, and Jenscare. Dr Leurent has received speaker and proctoring honoraria from Edwards Lifesciences and Abbott Medical. Dr Hausleiter has received research support and speaker honoraria from Edwards Lifesciences. All other authors have reported that they have no relationships relevant to the contents of this paper to disclose.
