# Supplementary material for: Transjugular Transcatheter Tricuspid Valve Replacement in Patients With Cardiac Implantable Electronic Devices
Source: JACC Asia. 2025 Aug 30;5(10):1260–9. doi: 10.1016/j.jacasi.2025.07.009 (PMC12790058; doi:10.1016/j.jacasi.2025.07.009)
Supplement: Supplemental Tables 1 and 2 [file mmc1.docx]

**Supplemental Table 1. Participating Centers**

| Centre | CIED cases | Total Cases |
| --- | --- | --- |
| St. Michael’s Hospital, University of Toronto, Toronto, Ontario,  Canada | 12 | 18 |
| Centre for Cardiovascular Innovation, St Paul's and Vancouver  General Hospital, Vancouver, BC, Canada | 11 | 26 |
| Department of Structural Heart Disease, Division of Cardiology,  Henry Ford Health System, Detroit, USA | 5 | 9 |
| Division of Cardiology, Department of Medicine and Therapeutics,  Prince of Wales Hospital, Chinese University of Hong Kong,  HKSAR, China; Central Medical, HKSAR, China | 3 | 10 |
| Department of Interventional Cardiology, Hospital Alvaro  Cunqueiro, Vigo, Spain; Instituto de Investigación Galicia Sur,  Servizo Galego de Saude, Vigo, Spain | 2 | 5 |
| Cardiology Department, Centre Cardiologique du Nord, Saint-  Denis, France | 2 | 2 |
| Medizinische Klinik und Poliklinik I, LMU Klinikum, LMU  München, Munich, Germany | 1 | 7 |
| Clinique Pasteur, Toulouse, France | 0 | 6 |
| The Heart Center, Rigshospitalet, Copenhagen, Denmark | 0 | 6 |
| Univ. Lille, Inserm, CHU Lille, Institute Pasteur de Lille, U1011-  EGID, F-59000 Lille, France | 0 | 2 |
| Department of Cardiology, University of Bern, Inselspital, Bern, Switzerland | 0 | 2 |
| Montefiore-Einstein Center for Heart and Vascular Care,  Montefiore Medical Center, Albert Einstein College of Medicine,  Bronx, New York | 0 | 2 |
| Department of Cardiology, Smidt Heart Institute, Cedars-Sinai  Medical Center, Los Angeles, California, USA | 0 | 1 |
| Department of Cardiology and Cardiovascular Surgery, Heart  Valve Center, Institut Cœur Poumon CHU de Bordeaux, France | 0 | 1 |
| Department of Cardiology, Complejo asistencial universitario de  Salamanca (CAUSA), Salamanca, Spain | 0 | 1 |
| Department of Cardiology, CHU Rennes, Inserm, LTSI-UMR 1099,  Univ Rennes 1, 35000 Rennes, France | 0 | 1 |
| Total | 36 | 99 |

**Supplemental Table 2. Transjugular TTVR in patients with or without CIEDs, excluding Leadless Pacemakers: Comparative Outcomes**

|  | CIED Excluding Leadless Pacemaker (N = 34) | No CIED (n = 63) | P Value |
| --- | --- | --- | --- |
| **Procedure Time, Min, mean+/-SD** | 152.8 +/- 52.4 | 125.5 +/- 46.9 | 0.125 |
| **TVARC intra-procedural success, %** | 91.2% (31/34) | 95.2% (60/63) | 0.420 |
| **Device and Procedural Complication, %** | 8.8% (3/34) | 3.2% (2/63) | 0.340 |
| Malposition of the device | 1 | 0 |  |
| Embolization of the device | 1 | 0 |  |
| Pericardial effusion before valve deployment | 1 | 0 |  |
| Anchor detachment | 0 | 1 |  |
| Incomplete extension of anterior leaflet graspers | 0 | 1 |  |
| **Need for in-hospital open heart surgery, %** | 8.8% (3/34) | 1.6% (1/63) | 0.122 |
| **Inpatient death, %** | 5.9% (2/34) | 4.8% (3/63) | >0.999 |
| Right heart failure | 1 (2.9%) | 2 (3.2%) |  |
| Gastrointestinal bleeding | 1 (2.9%) | 0 |  |
| Tamponade | 0 | 1 (1.6%) |  |
| **In hospital bleeding complication, %** | 5.9% (2/34) | 7.9% (5/63) | >0.999 |
| **In hospital Myocardial infarction, %** | 0% (0/34) | 0% (0/63) | >0.999 |
| **In hospital stroke, %** | 0% (0/34) | 0% (0/63) | >0.999 |
| **In hospital new onset renal failure requiring dialysis, %** | 5.9% (2/34) | 0% (0/63) | 0.120 |
| **Inpatient requiring new permanent pacemaker, %** | 0% (0/34) | 4.8% (3/63) | 0.550 |
| **30-day TVARC clinical success, %** | 89.7% (26/29) | 90.6% (48/53) | >0.999 |
| **30-day Death, %** | 5.9% (2/34) | 4.8% (3/63) | >0.999 |
| **30-day NYHA improvement >=1 grade, %** | 75.0% (18/24) | 81.6% (40/49) | 0.547 |
| **30-day NYHA I/II, %** | 83.3% (20/24) | 87.8% (43/49) | 0.459 |
| **30-day <=Mild TR (<=1+), %** | 82.8% (24/29) | 84.9% (45/53) | >0.764 |
| **TR reduction >=1 grade, %** | 100% (29/29) | 98.1% (52/53) | >0.999 |
| **TR reduction >=2 grade, %** | 96.6% (28/29) | 98.1% (52/53) | >0.999 |
| **30-day any PVL, %** | 65.5% (19/29) | 24.5% (13/53) | <0.001 |
| **30-day >=severe PVL, %** | 10.3% (3/29) | 7.5% (4/53) | 0.694 |
| **Repeat TV intervention at follow-up, %** | 12.5% (3/24) | 3.3% (2/60) | 0.138 |
| Heterotopic TTVR for severe PVL due to septal Anchor detachment | 1 | 0 |  |
| PVL Plug | 2 | 1 |  |
| TVR for severe PVL due to septal anchor detachment | 0 | 1 |  |
| **RV Lead Dysfunction requiring Revision at Follow-up, %** | 0% (0/34) | N/A | N/A |
| **RV Lead Dysfunction requiring Reprograming at Follow-up, %** | 10.0% (2/20) | N/A | N/A |
